# Biomarkers in ANCA associated vasculitis: clinical utility, pitfalls and their role in the outcomes assessment

**DOI:** 10.3389/fimmu.2025.1616837

**Published:** 2025-09-22

**Authors:** Chiara Marvisi, Caterina Ricordi, Cecilia Catellani, Stefania Croci, Francesco Muratore, Carlo Salvarani

**Affiliations:** ^1^ Rheumatology Unit, Azienda Unità Sanitaria Locale - IRCCS di Reggio Emilia, Reggio Emilia, Italy; ^2^ University of Modena and Reggio Emilia, Modena, Italy; ^3^ Clinical Immunology, Allergy and Advanced Biotechnologies Unit, Azienda Unità Sanitaria Locale - IRCCS di Reggio Emilia, Reggio Emilia, Italy

**Keywords:** biomarkers, ANCA, vasculitis, disease activity, outcome

## Abstract

Antineutrophil cytoplasmic antibody (ANCA)-associated vasculitides (AAV) are rare autoimmune diseases with multisystemic organ involvement. Their pathogenesis remains incompletely understood, and reliable biomarkers for disease activity and treatment response are lacking.

This review explores current and emerging biomarkers in AAVs to identify disease phenotypes and activity, aiming to optimize management and immunosuppressive treatment. Serological, cellular, and urinary biomarkers will be discussed, focusing on their current utility in clinical practice for assessing disease activity, damage related to the disease, and prognosis. Promising biomarkers and novel methodologies for detecting future biomarkers will also be briefly discussed.

## 1 Introduction

Antineutrophil cytoplasmic antibody (ANCA)- associated vasculitis (AAV) is an immune-mediated disorder affecting small—to medium-sized blood vessels. It encompasses granulomatosis with polyangiitis (GPA), microscopic polyangiitis (MPA), and eosinophilic granulomatosis with polyangiitis (EGPA) (1).

The etiology of AAV remains unclear, but it is believed to result from infectious and environmental triggers acting on a genetically susceptible background, leading to loss of tolerance to neutrophilic proteins, particularly proteinase 3 (PR3) and myeloperoxidase (MPO) (2, 3).

AAVs are rare diseases with a prevalence of approximately 200–400 cases per million. Increased awareness and the availability of non-invasive diagnostic tests have contributed to a rising incidence over time (4). The clinical presentation of AAVs varies widely. MPA predominantly affects the kidneys, while GPA and EGPA are characterized by extravascular granulomatous inflammation, primarily affecting the respiratory tract (5).

Severe AAV occurs with capillaritis, most commonly presenting as glomerulonephritis (GN) or alveolar hemorrhage (6).

Despite advances in treatment, there remains a critical need for biomarkers to assess disease activity, relapse risk, response to treatment, and end-organ damage. Various clinical tools aid in disease evaluation in everyday practice, including the Birmingham Vasculitis Activity Score (BVAS) version 3 and AAV- Patient Reported Outcomes (AAV- PRO) for disease activity, the Vascular Damage Index (VDI) for vasculitic damage, and the Five Factor Score (FFS) as a prognostic index, though the latter is prevalently used in EGPA (7–10).

As in other diseases, histopathological assessment remains a key but invasive biomarker for diagnosing AAV and evaluating its activity, extent, and prognosis, typically through kidney biopsy. The histopathological classification of ANCA-associated GN significantly impacts patient outcomes: the focal class (≥50% normal glomeruli) is associated with the most favorable prognosis, while the sclerotic class (≥50% globally sclerosed glomeruli) corresponds to the poorest prognosis (11).

However, renal biopsy is not always feasible and may pose an increased risk of bleeding for some patients. Furthermore, some patients may not show kidney involvement but have other extra-renal manifestations, such as purpura, lung infiltrates, or nasal crusting. In these cases, the affected organ should be biopsied, but the diagnostic value of histological examination of organs other than the kidney remains limited (12).This highlights the need for non-invasive biomarkers, especially in the era of personalized medicine, where reliable biomarkers are essential for predicting outcomes and guiding treatment decisions (13).

**Figure 1:**
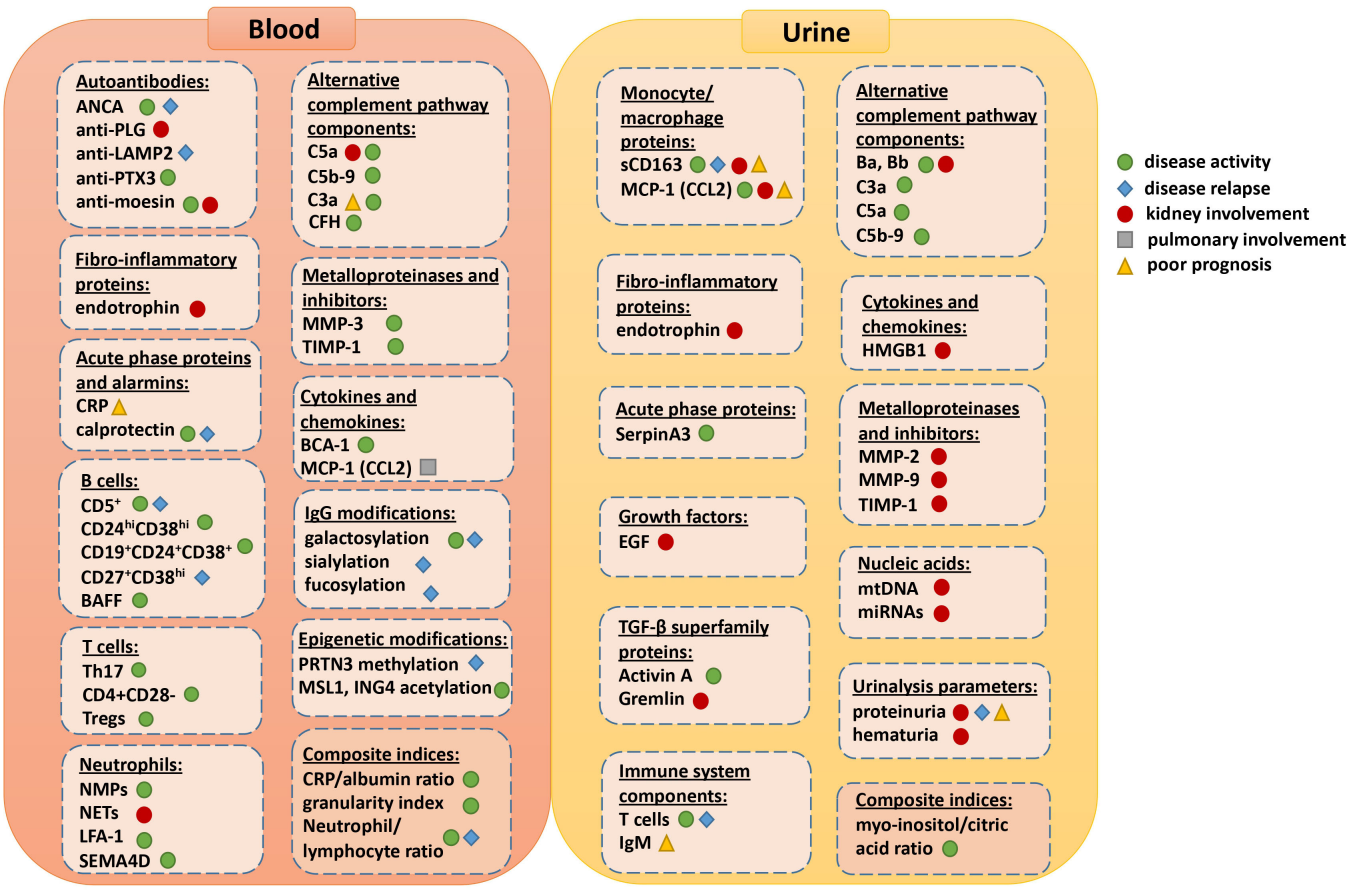
Visual summary of all the available blood and urine biomarkers and their current or potential role for assessing disease activity, relapse, prognosis or specific organ involvement.

Current research explores various serum and urinary biomarkers as potential alternatives to histopathological examinations for assessing disease activity, damage, and prognosis, as summarized in **Table 1**, **2** and **Figure 1**. However, most of these biomarkers require validation, and studies often show conflicting results. This review will examine the existing evidence, highlighting the potential clinical utility and limitations of non-invasive biomarkers under investigation.

**Table 1:** Summary of the clinical utility of the serum biomarkers.

Biomarker	Diagnosis	Disease activity	Prognosis	Damage	Specific organ involvement	Clinical applicability
PR3 ANCA	**√**	**X**	**√**	**X**	**√**	**√**
MPO ANCA	**√**	**X**	**√**	**X**	**√**	**√**
Anti PLG	**X**	**X**	**√**	**X**	**√**	**X**
Anti LAMP2	**X**	**√**	**X**	**X**	**√**	**X**
Anti Moesin	**X**	**X**	**X**	**X**	**√**	**X**
Anti PTX 3	**X**	**√**	**X**	**X**	**X**	**X**
C3 and C4	**X**	**√**	**√**	**X**	**X**	**√**
ESR and CRP	**X**	**√**	**X**	**X**	**X**	**√**
Calprotectin	**X**	**X**	**√**	**√**	**X**	**√**
MMP3	**X**	**√**	**X**	**X**	**X**	**X**
CXCL13	**X**	**√**	**X**	**X**	**√**	**X**
TIMP1	**X**	**√**	**X**	**X**	**X**	**X**
Endothropin	**X**	**X**	**X**	**X**	**√**	**X**
CCL2	**X**	**X**	**X**	**X**	**√**	**X**
CD27^+^CD38^hi^ B-cells	**X**	**X**	**√**	**X**	**X**	**√**

ANCA, anti-neutrophyls cytoplasmic antibody; CRP, C reactive protein; ESR, erythrocyte sedimentation rate; LAMP2, lysosomial associated membrane protein 2; MCP-1, monocyte chemoattractant protein-1; MMP3, matrix metalloproteinase 3; MPO, myeloperoxidase; PLG, plasminogen; PR3, proteinase 3; PTX3, pentraxin 3; TIMP1, tissue inhibitor of metalloproteinase 1. Bold characters were used to better visualize the content of the tables.

**Table 2:** Summary of the clinical utility of the urinary biomarkers.

Biomarker	Diagnosis	Disease activity	Prognosis	Damage	Clinical applicability
sCD163	**X**	**√**	**√**	**X**	**X**
MCP-1	**X**	**√**	**X**	**X**	**X**
CCL2	**X**	**√**	**√**	**X**	**X**
Proteinuria	**X**	**√**	**X**	**√**	**√**
Bb	**X**	**X**	**√**	**X**	**X**
HMGB1	**X**	**√**	**X**	**X**	**X**
EGF	**X**	**√**	**√**	**X**	**X**
Endothropin	**X**	**x**	**X**	**X**	**X**
Serpin A3	**X**	**X**	**X**	**√**	**X**
Activin A	**X**	**√**	**X**	**X**	**X**
Gremlin	**X**	**√**	**X**	**X**	**X**
T cells	**X**	**√**	**√**	**X**	**X**
IgM	**X**	**X**	**√**	**X**	**X**
mtDNA	**X**	**X**	**√**	**X**	**X**
MMPs*	**X**	**X**	**√**	**X**	**X**
Myoinositol:citric acid ratio	**√**	**X**	**X**	**X**	**X**

*: MMP-2, MMP-9, and TIMP-1.

EGF, epidermal growth factor; HMGB1, High-mobility group box; MCP-1, monocyte chemoattractant protein; sCD163, soluble CD163. Bold characters were used to better visualize the content of the tables.

## 2 Serum biomarkers

### 2.1 ANCA

#### 2.1.1 ANCA measurement methods

**Figure 2:**
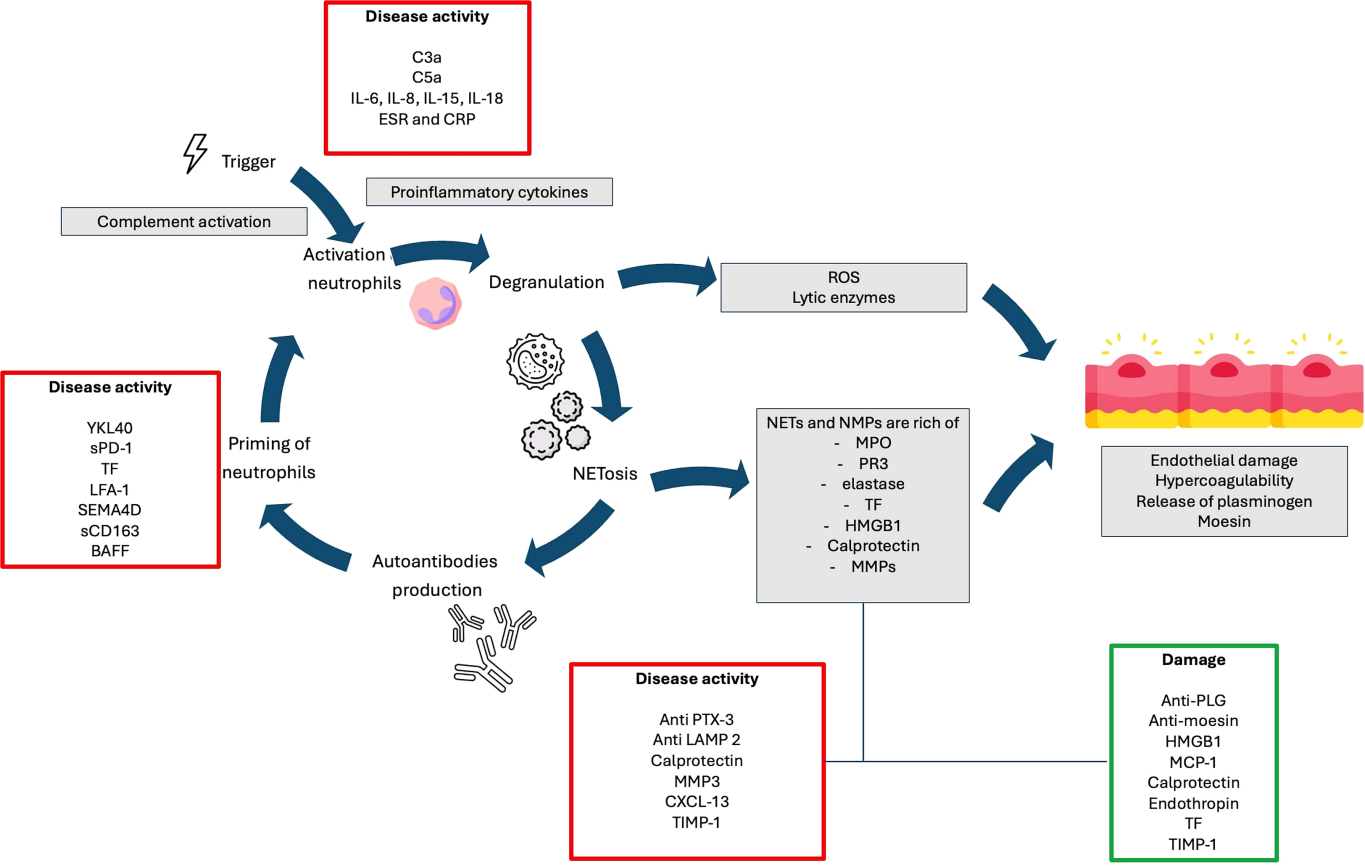
Potential pathogenic pathways involved in the development of AAV and their relationships with biomarkers.

ANCAs are autoantibodies, primarily of the IgG1 and IgG4 subclasses, targeting constituents of primary granules of neutrophils and monocyte lysosomes. They are implicated in AAV pathogenesis by excessively activating cytokine-primed neutrophils, leading to the release of reactive oxygen species, proteolytic enzymes, and the formation of neutrophil extracellular traps (NETs), ultimately contributing to endothelial damage (**Figure 2**) (14–17).

Various methods exist for ANCA detection, but high-quality antigen-specific immunoassays are now preferred due to their superior diagnostic accuracy (18, 19). Indirect immunofluorescence (IIF) may serve as a screening tool or in cases of high clinical suspicion with negative immunoassay results. However, IIF has several limitations, including a lack of standardization, subjective qualitative interpretation, and low specificity.

Among several antigenic targets, PR-3-ANCA and MPO-ANCA are the most clinically relevant (20, 21). They represent sensitive and specific markers for AAV diagnosis in the appropriate clinical context and are included in the latest AAV classification criteria (22–24). However, a subset of AAV patients remains ANCA negative, including 5-10% of GPA/MPA cases and up to 70% of EGPA cases (3, 25).

On IIF, anti-PR3 antibodies typically produce a cytoplasmic staining pattern (cANCA) in nearly 90% of cases, while anti-MPO antibodies generally result in a perinuclear staining pattern (pANCA) with nuclear involvement in about 40% of cases (26). Perinuclear fluorescence without nuclear extension, particularly in MPO-ANCA positive cases, can also occur in conditions beyond AAV, including inflammatory bowel diseases (IBDs), autoimmune hepato-biliary diseases, anti-glomerular basement membrane disease, and other systemic autoimmune diseases (e.g, systemic lupus erythematosus, rheumatoid arthritis), and infections (27–31). Additionally, minor ANCAs often produce a pANCA or an atypical mixed pANCA/cANCA staining pattern. These atypical ANCAs target antigens such as enolase, azurocidin, elastase, cathepsin G, lactoferrin, and defensin. They are primarily associated with AAV secondary to other causes, particularly drug-induced vasculitis (32).

#### 2.1.2 Clinical significance of ANCA

Clinically, AAV patients with pauci-immune necrotizing crescentic glomerulonephritis typically test positive for either PR3-ANCA or MPO-ANCA (33). Regarding extra-renal manifestations, MPO-ANCA is more frequently associated with nasal polyposis and interstitial lung disease (ILD), which can progress to lung fibrosis (34). In contrast, PR3-ANCA is linked to an increased risk of granulomatous inflammation affecting the ear, nose, and throat (ENT) tract, as well as the lower respiratory tract, manifesting as tracheobronchial stenosis, excavated nodules, and life-threatening diffuse alveolar hemorrhage (35–39).

While ANCA testing is well established for diagnostic purposes, its role in monitoring disease activity during follow-up remains uncertain (40–48).

It is well-documented that PR3-ANCA-positive patients have a higher risk of relapse compared to MPO-ANCA positive patients (35, 49–54). A persistently elevated or rising PR3-ANCA titer has been associated with an increased risk of relapse within one year in GPA and MPA. Conversely, patients who achieve ANCA negativity with treatment tend to have a lower risk of relapse (53, 55–58).

The secondary analysis of the RAVE trial, which compared cyclophosphamide versus rituximab for induction treatment in AAV patients, demonstrated a significant association between relapse risk and rising PR3-ANCA titers, particularly among patients with major organ involvement treated with rituximab. In this cohort, involving only patients with a PR3 positivity, the increase in PR3-ANCA titers was associated with a higher risk of relapse (HR 2.24; 95% CI 1.24–4.08, p = 0.008), with an even stronger association for severe relapse (HR 4.57; 95% CI 2.16–10.37, p < 0.001) (55). However, ANCA titers did not consistently correlate with disease activity, as 17% (16/93) of patients exhibited a negative ANCA status despite active disease, while 20.4% (19/93) of patients with rising (or elevated) ANCA titers did not experience a relapse. Moreover, no definitive cutoff value for ANCA increase was established (55). Similarly, a Japanese cohort study of 271 anti-MPO-positive patients found that persistent or re-emerging MPO-ANCA after induction treatment was associated with an increased relapse risk (OR 26,2 CI 8,2-101; p < 0.001). However, 73% of patients with MPO-ANCA reappearance did not subsequently relapse, further underscoring the limitations of ANCA titers as a sole predictor of disease activity (59).

Longitudinal ANCA testing (either MPO or PR3) has been postulated as a predictor of relapse, especially in AAV patients with renal involvement or severe non-renal disease (48, 59, 60). A recent systematic review and meta-analysis of 20 studies found that an ANCA rise more frequently preceded disease relapse within 6 months (OR 3.65, 95% CI 1.66–8.03) than within 12 months (OR 2.88, 95% CI 1.21–6.88). However, ANCA titers were not indicative of an ongoing relapse at the time of testing (OR 0.13, 95% CI 0.03–0.53) (61).

Despite these findings, using an increase in ANCA titers to guide maintenance treatment decisions did not significantly increase the risk of relapse compared to regular maintenance therapy at fixed 6-month intervals (62, 63). This suggests that while ANCA trends may provide some prognostic insight, they are not yet reliable enough to indicate treatment adjustments alone.

In summary, the latest recommendations on AAV management suggest that ANCA titers should be considered as part of the clinical evaluation of AAV patients (18). However, the absence of definite cutoff values, the variability of testing methods, and their suboptimal predictive value for future relapses limit the utility of ANCA alone as biomarkers for guiding therapeutic decisions (56, 64–68).

### 2.2 Other autoantibodies

Other antibodies that may be co-expressed with or independent of ANCA include anti-tissue plasminogen (anti-PLG), anti-lysosomal-associated membrane protein-2 (anti-LAMP2), anti-moesin, and, anti-pentraxin 3 (**Table 1**) (69–73).

Plasminogen (PLG) is a key protein in the fibrinolytic system, and its impairment can lead to thrombotic events. Anti-PLG autoantibodies have been found in approximately ¼ of ANCA-positive AAV cohorts (73). In a study of 74 AAV patients, anti-PLG autoantibodies were found in 18 cases (24.3%) and were associated with more severe renal disease, as evidenced by a higher percentage of fibrinoid necrosis (p < 0.05) and cellular crescents (p < 0.001) compared to seronegative patients (73).

Antibodies directed against lysosomal-associated membrane protein (anti-LAMP2) are co-expressed on neutrophils in a significant proportion (90-91%) of ANCA-positive AAV patients. Although they do not show a clear correlation with disease activity at diagnosis, their behavior closely mirrors that of ANCA during immunosuppressive treatment (72). The titers of those antibodies, targeting a heavily glycosylated membrane protein involved in neutrophil regulation tend to decrease rapidly following immunosuppressive treatment and rise again during disease relapse (72, 74).

Moesin is a heparin-binding protein expressed on cellular membranes, including glomerular endothelial cells. Anti-moesin autoantibodies have been observed in sera of mice that spontaneously develop MPO-ANCA-associated rapidly progressive glomerulonephritis, as well as in patients with MPO-AAV (75). By binding to moesin, those autoantibodies lead to the activation of glomerular endothelial cells and of neutrophils (69). Thus, they could serve for identifying patients at risk for more severe renal involvement, especially in MPO+ AAV patients.

Finally, anti-pentraxin3 (anti-PTX3) antibodies, which produce a distinct IIF pattern on fixed granulocytes (distinct from pANCA, cANCA and atypical ANCA patterns), have been detected in nearly 1/3 of AAV patients, particularly during active disease (70, 71). Anti-PTX3 are thought to contribute to the disease mechanism by interfering with opsonization and removal of apoptotic cells, and by regulating complement activation.

Overall, data on these secondary antibodies are limited and are primarily associated with disease phenotype and complex pathogenetic mechanisms that may involve interactions with ANCAs. Among them, only anti-LAMP antibodies have been studied in relation to treatment response, showing a behavior similar to ANCAs. However, the clinical significance and predictive value of these autoantibodies in AAVs are yet to be fully defined.

### 2.3 Complement

#### 2.3.1 Role of complement in the pathogenesis of AAV

The role of complement in AAV has historically been considered minor due to the “pauci-immune” pattern observed in renal biopsies (76). Circulating levels of C3 and C4 are usually normal in patients with AAV. However, recent studies have elucidated complement’s role in mediating endothelial injury.

In particular, the alternative pathway appears to play a crucial role in AAV pathogenesis, aligning with the understanding that AAV is not primarily driven by immune complexes, which predominantly activate the classical pathway (77). All complement pathways lead to the generation of C5, which is then cleaved into C5a, an anaphylatoxin, and C5b, which facilitates the assembly of the lytic membrane attack complex (MAC, also known as C5b-9).

In the common pathway, C5a and not MAC plays a pivotal role in ANCA-mediated lesions (78). Serum levels of fragments from the common pathway (C3a and C5a) and the alternative pathway (factor B) have been found to be higher in patients with active disease compared to those in remission. Additionally, factor B correlates with the proportion of cellular crescents, disease activity assessed by the BVAS, and acute phase reactants such as the erythrocyte sedimentation rate (79). A study by Moiseev et al. evaluated complement levels in 59 patients with AAV and 36 healthy volunteers, combining results with four previous studies to analyze a sample of more than 220 patients and 120 controls (80–83).

The meta-analysis confirmed complement activation in active AAV, showing significantly higher levels of C5a, MAC, and factor B among AAV patients with active disease compared to those in remission and healthy controls. Additionally, analysis of the initial cohort found no significant differences in complement product levels between MPO ANCA-positive and PR3 ANCA-positive patients, MPA and GPA, severe and non-severe vasculitis, or forms with predominant granulomatous or vasculitic manifestations.

Recently, circulating immune complexes (CICs) have been found positive in 65% of a total of 20 patients with a diagnosis of MPA and renal involvement. Their levels were positively correlated with C5a and C5b-9 levels and negatively correlated with levels of the classical pathway kit. These findings may suggest that the complement is activated in AAV also via the classical pathway (84).

#### 2.3.2 Role of complement in assessing disease activity, damage and prognosis

Several retrospective studies have examined circulating C3 levels and their relationship to patient outcomes. Up to 35% of patients present with low C3 levels at initial diagnosis, which correlates with more severe disease and poorer renal function at presentation (85, 86). Prognostically, low C3 levels have been associated with worse renal and overall survival (87–89).

While the role of circulating complement fragments is well established in assessing systemic disease activity in AVV, various studies have investigated their potential to predict histological findings in renal biopsies. Gou et al. found that glomerular Bb deposition in AAV patients correlated with crescent formation, interstitial infiltrates, interstitial fibrosis, and tubular atrophy, while inversely correlating with the proportion of normal glomeruli (90). Additionally, urinary Bb, C3a, C5a, and soluble C5b-9 levels were significantly elevated in active renal disease. However, these findings were not confirmed in other cohorts. Moiseev et al. reported that, in a small group of patients with kidney biopsies, factor B levels correlated with glomerular C3 deposition, whereas other complement components did not show significant associations with histological parameters (83).

Recently, Matsuda et al. evaluated prognostic biomarkers for end-stage renal disease in 74 patients with MPA. They found in the multivariate analysis and in the receiver operating characteristic analysis that high serum creatinine (cut-off value of 3.54 mg/dl) and high serum C4 levels (cut-off of 29.6 mg/dl) were the strongest risk factors for end-stage renal disease (91). These findings suggest that also C4 levels may be a prognostic tool in MPA with kidney involvement.

The predictive potential of circulating complement levels in relapsing disease remains poorly defined, and studies in this area are limited. Nonetheless, research into complement levels has significantly enhanced our understanding of the C5a axis, which has translated into therapeutic advances. For example, avacopan, an orally administered inhibitor of the C5a receptor (C5aR), blocks C5a-induced activation without affecting its binding to the C5a-like receptor 2, which acts as a scavenger and is associated with milder renal disease (92). In the ADVOCATE trial, a large, randomized trial comparing avacopan plus placebo with standard glucocorticoid therapy, both combined with cyclophosphamide or rituximab, avacopan was found to be non-inferior to prednisone taper for remission at 26 weeks and superior in achieving sustained remission at 52 weeks (93). Additionally, IFX-1, a monoclonal antibody targeting C5a, is currently under investigation in a Phase II trial (NCT03712345), representing another promising approach for managing AAV by modulating the C5a pathway (94).

### 2.4 Other serological biomarkers

**Table 1** and **Figure 1** summarize other serological biomarkers that may be used in clinical practice. Acute phase reactants include traditional inflammatory markers, such as the erythrocyte sedimentation rate (ESR) and C-reactive protein (CRP), along with others like calprotectin, hepcidin, and procalcitonin. While these markers are used in clinical practice to monitor disease activity in AAV with multisystem involvement, they lack the specificity needed to reliably differentiate active disease from remission or permanent damage, and to distinguish between relapsing and non-relapsing disease (95–102). A large cross-sectional study even confirmed a poor correlation between the Birmingham Vasculitis Activity Score (BVAS) and CRP (103).

Among these markers, the calprotectin heterodimer (S100A8/S100A9) has recently attracted interest as a potential surrogate for vasculitis damage. Calprotectin, a ligand for toll-like receptor-4, is abundantly expressed in neutrophils and monocytes and is released following their interaction with activated, inflamed endothelium. Once secreted, the calprotectin complex binds to activated endothelial cells via heparan sulfate proteoglycans, triggering proinflammatory effects, and promoting further recruitment of leukocytes, especially neutrophils. This cascade contributes to the impairment of endothelial integrity and initiates both caspase-dependent and caspase-independent cell death mechanisms, leading to apoptosis, necrosis, and NETosis, thereby positioning calprotectin as a potentially critical mediator of tissue damage in AAV (104).

Several other blood-based biomarkers, including cytokines and chemokines, show promise in distinguishing active AAV from remission. For instance, Monach et al. evaluated multiple serum proteins in 186 patients with active, severe GPA or MPA participating in the RAVE trial. Matrix metalloproteinase-3 (MMP-3) (AUC 0.89; cut-off 38 ng/mL, sensitivity 82%, specificity 88%), CXCL13 (AUC 0.86; cut-off 70 pg/mL, sensitivity 77%, specificity 85%), and tissue inhibitors of metalloproteinases-1 (TIMP-1) (AUC 0.83; cut-off 270 ng/mL, sensitivity 78%, specificity 82%) effectively distinguished between active disease and remission. Furthermore, during follow-up, serum levels of CXCL13, IL-6, IL-8, IL-15, IL-18BP, and MMP-3 were significantly associated with AAV disease activity. Among these, IL-8, IL-15, and IL-18BP emerged as the most promising predictors of disease flare, with these findings being independent of age, sex, ANCA specificity, treatment, and whether the disease was new-onset or relapsing (102). Additional biomarkers, such as serum chitinase-3-like 1 protein (YKL40) and soluble programmed cell death protein 1 (sPD-1) have been observed at elevated levels in patients with severe AAV. Both YKL40 and sPD-1 demonstrated significant correlations with the Five Factor Score and the BVAS (105, 106).

Additional biomarkers have been investigated as predictors of organ damage, complementing the gold standard of renal biopsy for assessing glomerular sclerosis and fibrosis (13). Serum endotrophin, a matriline derived from collagen type IV, has shown strong correlations with renal function in AAV patients with kidney involvement. In a study of 47 patients, endotrophin levels correlated positively with serum creatinine (r=0.81, p<0.0001) and inversely with estimated glomerular filtration rate (eGFR) (r=−0.82, p<0.0001). Patients with more advanced chronic kidney disease (CKD stages 3, 4, 5) had significantly higher endotrophin levels compared to those with CKD stage one. Moreover, serum endotrophin levels showed significant correlations with the extent of renal fibrosis and baseline endotrophin levels were predictive of CKD progression over a 3-year follow-up period (AUC = 0.807, 95% CI 0.615 to 0.931; p<0.001) (107).

In lung biopsies from patients with MPA-associated interstitial lung disease (ILD), immunohistochemistry revealed intense CCL2 staining in CD68+/CD163+ macrophages and metaplastic epithelial cells. Correspondingly, serum CCL2 levels have been investigated as a non-invasive biomarker for ILD. High serum CCL2 levels were associated with ILD presence, and baseline levels correlated with total lung fibrosis scores at a 1-year follow-up (r=0.51, p=0.005) (108). In addition, initial levels of CXCL13 correlated with the total ground-glass opacity (r = 0.53). The total fibrosis score correlated well not only with initial levels of CXCL13 (r = 0.38), but also with initial levels of IL-13 (r= 0.35). The presence of CXCL13-producing B cells in the lymphoid follicles in the interstitium of patients with MPA-ILD was also confirmed by immunohistochemical analysis, hence suggesting a potential role as a prognostic biomarker and a surrogate of lung biopsy (109).

## 3 Cellular biomarkers

In this section we will discuss current data about the value as biomarkers of immune cells of the innate and adaptive immunity in AAV.

### 3.1 B cells

B lymphocytes play a central role in the pathogenesis of AAV. Autoreactive B cells produce ANCAs that can activate neutrophils, leading to damage in small blood vessels, such as in the renal glomeruli and respiratory tract. The frequency of antigen-specific plasmablasts and plasma cells is increased in AAV patients. Besides antibody production, B lymphocytes display an imbalanced subset distribution, dysregulated cytokine production, and can present antigens to T cells. Studies on potential changes in transitional B cells and memory B cells in the peripheral blood of AAV patients are currently inconsistent. Naïve B cells have been found to be increased in number and more responsive to B cell receptor stimulation. B1 cells and marginal zone B cells are reduced in circulation during active AAV (110).

The key role of B cells in AAV pathogenesis, is also evidenced by the efficacy of Rituximab, a CD20-targeted therapy, in treating the disease. The therapeutic effectiveness of rituximab is achieved through B-cell depletion, leading to a decrease in pathogenic ANCAs (anti-PR3 and anti-MPO). Rituximab primarily targets peripheral CD20+ B cell subsets. However, long-lived plasma cells and CD20-negative B cell subsets are not affected and can remain a source of ANCAs, which is a potential limitation of rituximab. Several studies have investigated whether B cell counts can serve as biomarkers for disease activity and predictors of relapse. Observational data, including findings from the follow-up of the RITUXVAS trial, indicated that B cell repopulation after Rituximab treatment was associated with a higher relapse rate (111, 112). In that study, B cells returned at a median of 12.6 months, and 30% of patients with B cell repopulation had a relapse, suggesting that sustained B cell depletion might be necessary to maintain remission (112). However, analyses from the RAVE, MAINRITSAN, and MAINRITSAN2 cohorts did not confirm these findings (62, 113, 114). In these studies, relapses were observed even in patients with undetectable B cell levels, and B cells were sometimes present during periods of quiescent disease. These conflicting results suggest that while B cells are clearly involved in AAV pathogenesis, their levels alone may not reliably predict disease relapse, and additional factors likely contribute to disease activity and flare.

Other B cell subpopulations have also been explored, particularly regulatory B cells (CD5+ and/or CD24+ and CD38+ B cells), that help downregulate B cell activity.

For example, the percentage of CD5+ B cells was found to be lower in patients with active AAV compared to those in remission or controls, and these levels increased during treatment-induced remission (115). Moreover, a downward trend in CD5+ B cells was associated with disease relapse (115). Similarly, the CD5+ subset of CD24hiCD38hi B cells was reduced in active AAV patients relative to controls and increased during remission, suggesting that these regulatory B cells may support long-term clinical remission (116). However, subsequent studies did not confirm the potential of CD5+ B cells to reliably anticipate disease relapse (117).

One study reported that the overall percentage of CD24hiCD38hi B regs was higher in patients in remission compared to controls, with no significant difference compared to active patients (116). In contrast, another study found lower percentages of these cells during remission compared to controls, and yet another observed that treated GPA patients had lower percentages of CD24hiCD38hi B regs compared to both untreated GPA patients and controls (118, 119).

In summary, while these findings indicate that regulatory B cells may be involved in the disease process, their precise role in AAV progression remains unclear and needs further investigation.

Regarding other B cell subtypes in relation to disease course, an increased frequency of CD27^+^CD38^hi^ B cells during remission has been associated with a higher risk of relapse in GPA patients. Specifically, those with <2.9% of circulating CD27^+^CD38^hi^ B cells had significantly higher relapse-free survival compared to patients with ≥2.9% (HR: 8.8; 95% CI 3.35-23.2) (119).

These findings suggest that CD27+CD38hi B cells could serve as a potential biomarker for predicting relapse risk in GPA (**Table 1**).

### 3.2 T cells

As in other autoimmune diseases, the T cell homeostasis in AAV is skewed toward memory and pro-inflammatory subsets. Among the most investigated are T helper 17 (Th17) and CD4+CD28neg T cells, both of which contribute to endothelial damage and granuloma formation. These cells serve as significant sources of pro-inflammatory cytokines, perpetuating immune-mediated tissue damage.

Th17 cells are a subset of CD4+ T cells that play a dual role in immune responses, contributing to both early pathogen defense and the development of chronic inflammation seen in autoimmune diseases, through the production of various cytokines. The primary cytokine product, IL-17, drives tissue inflammation and neutrophil recruitment (120). A reciprocal interaction exists between Th17 cells and neutrophils, where each can enhance the recruitment and activation of the other at sites of inflammation (121).

Studies investigating Th17 cells in AAV have yielded mixed results regarding their association with disease activity. Increased Th17 cell percentages were observed in both active and inactive GPA patients compared to controls, with similar results in EGPA (122, 123). Additionally, peripheral blood stimulation with PR3 led to a significant Th17 cell increase in ANCA-positive patients compared to ANCA-negative patients and healthy controls (124). Elevated levels of the Th17-associated chemokine CCL20 were also detected in active AAV (125). However, other studies failed to confirm a Th17-skewed profile in AAV, possibly due to T cells’ plasticity, which can shift phenotypes in response to environmental stimuli (126).

CD28 expression on T cells was found to be lower in GPA patients compared to controls, with the percentage of CD28neg T cells positively correlating with disease extent (127). The CD4+CD28neg T cell subset was expanded in AAV patients compared to controls but did not correlate with BVAS (128). Phenotyping of CD4+CD28neg T cells in peripheral blood of GPA patients revealed expression of CD57 (also expressed by NK cells), CD18 (β2-integrin), and perforin. These cells were capable of producing high levels of TNFα and INFγ within granulomatous lesions, suggesting a cytotoxic role that may contribute to granuloma formation through cytokine secretion (129).

Regulatory T cells (Tregs) play a crucial role in suppressing inflammation, and their dysfunction has been implicated in AAV. Studies have consistently shown reduced Treg activity in AAV, with Treg frequencies closely related to inflammatory status and drug-free remission. Specifically, the Treg population was markedly reduced in active AAV patients compared to healthy subjects and was negatively associated with BVAS and relapse rates, highlighting their potential role in disease modulation (128, 130).

Interestingly, while Treg cells were increased in GPA patients in remission, their immunosuppressive function was diminished, potentially contributing to disease relapses (131). Furthermore, an increased frequency of Tregs was observed in active AAV patients compared to both the same patients in remission and healthy controls, yet their function remained impaired during active disease (132). Notably, the frequency of Tregs was reduced in AAV patients receiving conventional therapies compared to controls but remained similar in those treated with rituximab, suggesting a potential B cell-dependent regulation of Tregs and highlighting the need for further investigations into immune cell interactions (133).

Evaluating the Th1/Th2 balance in GPA and MPA patients could aid in monitoring disease progression and better defining the disease (134). Cytokines secreted by Th1 cells are involved in recruiting and activating neutrophils and macrophages at sites of vascular inflammation. In GPA, localized disease was mainly characterized by a Th1 pattern, which shifted to a Th0/Th2 T cell profile as the disease became more widespread. Additionally, a Th1 cytokine pattern, primarily IFNγ, was predominant in granulomatous inflammation observed in biopsied nasal mucosal tissue, bronchoalveolar lavage, and peripheral blood samples from GPA patients (135). A Th1 response can enhance neutrophil activation in the early stages of the disease, contributing to disease progression (136). T cell involvement in MPA is less well studied, but a prominent Th2 cell pattern has been observed in MPA patients with interstitial lung disease compared to those without, suggesting that Th2 cells may be involved in specific organ manifestations (109). Ultimately, Th2 cells play a central role in EGPA pathogenesis, inducing eosinophilic inflammation through the production of cytokines such as IL-4, IL-5, and IL-13, which promote eosinophil activation and survival.

Overall, the evidence regarding T cell subsets remains controversial and is largely derived from small cohorts. Therefore, further research is needed to determine whether T cells could serve as reliable biomarkers in peripheral blood.

### 3.3 Neutrophils

Neutrophils play a well-established role in the pathogenesis of AAV. In particular, the release of neutrophil-derived microparticles (NMPs) and neutrophil extracellular traps (NETs) by ANCA-activated neutrophils has highlighted their potential pathogenetic significance. Both NMPs and NETs contribute to endothelial injury and thrombin activation. Additionally, they may serve as a link between innate and adaptive immunity by presenting ANCA target antigens, PR3 and MPO. NMPs are submicron membranous vesicles that can exert proinflammatory and pro-coagulant stimuli during AAV. They are secreted by activated or apoptotic neutrophils. On the other hand, NETosis is a unique form of neutrophil cell death in which granule contents are extruded into the extracellular space along a mesh-like network or net. NETs consist of dsDNA, citrullinated histone peptides and azurophilic granule proteins such as MPO and neutrophil elastase (10.1038/s41577-022-00787-0). **Figure 2** summarizes the pathogenic roles of NMPs and NETs, as well as their implications as biomarkers of disease activity (137).

The release of NMPs is triggered by both polyclonal ANCAs isolated from AAV patients and chimeric PR3-ANCA (138). Studies indicate that NMPs can induce MPO-mediated vascular endothelial cell damage, an effect that can be reversed by the removal of NMPs or the administration of antioxidants (138, 139). Patients with active AAV exhibited increased circulating NMPs compared to those with inactive disease and healthy controls (138, 140). Additionally, AAV patients demonstrated higher expression of complement C3a and C5a on NMPs than controls, with even greater levels observed in patients with renal involvement (81). Notably, the expression of C3a and C5a on NMPs correlated with BVAS, reinforcing their potential as biomarkers of disease activity (81).

Peripheral blood neutrophils from patients with active disease displayed elevated levels of neutrophil tissue factor (TF), a key trigger of venous thrombosis, which may contribute to hypercoagulability in AAV. These neutrophils released TF- expressing NETs and NMPs detectable in serum (140). This was further supported by the release of TF- expressing NETs and NMPs from control neutrophils treated *in vitro* with sera from patients with active AAV. The effects were not observed after IgG depletion in sera but were evident when isolated IgG from patients with active disease was used. Both total NMPs and TF-expressing NMPs correlated with disease activity in AAV (140). Additionally, the expression levels of PTX3, HMGB1, and TWEAK, promising biomarkers of inflammatory processes, were higher in NMPs from active AAV patients compared to controls. Furthermore, levels of HMGB1, PTX3, and NMPs were elevated in active patients relative to those in remission, and the expression levels of HMGB1 and PTX3 positively correlated with disease activity (141). However, the clinical applicability of these findings requires further clarification.

Normally, NETs play a protective role against pathogens but can also adhere to the endothelium and cause significant damage (142). Treatment with serum IgG from MPA patients increased NET formation and decreased NET degradation in neutrophils from healthy volunteers compared to IgG from controls (142, 143). Moreover, the ability to form NETs was correlated with disease activity in MPA patients (143). However, this aspect is still debated, as other authors have reported that, in PR3-ANCA positive patients, an increase in NET production was not correlated with ANCA levels (142). In light of this evidence, it can be hypothesized that the combination of other factors, such as pro-inflammatory cytokines and damage-associated molecular patterns (DAMPs), may be involved in this process.

Serum NET levels, including cell-free DNA, citrullinated-histone H3-DNA, and MPO-DNA complexes, were found to be elevated in AAV patients compared to controls. However, no significant differences were observed between active AAV patients and those in remission, and there were no correlations with clinical parameters such as CRP and BVAS (144). These findings suggest that NET levels may not be reliable biomarkers for disease activity in AAV.

Additionally, NETs were observed to be deposited around sites of fibrinoid necrosis in the kidney, as well as in thrombi and in glomerular crescents in MPA patients, indicating their potential pathogenic role in the clinical manifestations of AAV (145, 146).

Finally, two proteins expressed by neutrophils, lymphocyte function-associated antigen-1 (LFA-1) and semaphorin 4D (SEMA4D), have been proposed as potential biomarkers of disease activity in AAV. LFA-1 is an integrin present in leukocytes that interacts with ICAM-1 and ICAM-2 on endothelial cells. The expression of LFA-1 on neutrophils was found to be higher in AAV patients compared to controls and patients with other diseases. Moreover, LFA-1 expression decreased during follow-up in patients who responded to treatment, while non-responders did not show this reduction. LFA-1 expression was also correlated with disease activity parameters (147). In contrast, the expression of SEMA4D was lower on neutrophils from AAV patients compared to healthy controls. However, serum concentrations of SEMA4D were elevated and positively correlated with both BVAS and blood neutrophil count. This increase in serum SEMA4D was attributed to increased proteolytic cleavage of cell-surface SEMA4D from stimulated neutrophils mediated by ADAM17. This process promoted inflammation in AAV by inhibiting the interaction of SEMA4D with endothelial cells, which led to the aberrant activation of neutrophils (148). However, further validation through larger cohort prospective studies is needed to confirm their potential as reliable disease biomarkers.

### 3.4 Others

Other potential cellular biomarkers include circulating endothelial cells and circulating endothelial microparticles, which are released from the vascular surface in response to various stimuli and are associated with endothelial injury (**Figure 2**). While some correlations with disease activity have been reported, these markers lack both specificity and standardized detection methods.

The granulocyte/granularity index, intended to distinguish between hyper- and hypogranular granulocytes, was found to be useful in identifying patients more likely to achieve remission when comparing different treatments (149). Additionally, the neutrophil-lymphocyte ratio has been associated with disease severity, risk of infection, and relapse, showing promising results (150–153). However, the neutrophil-lymphocyte ratio is not specific for AAV.

## 4 Urinary biomarkers

Urinary biomarkers are being investigated as potential surrogates for renal biopsy. While they offer a non-invasive method to assess disease activity, the lack of prospective studies limits their clinical application. This section will review the available data on the correlations of these biomarkers with disease activity, renal damage, and response to treatment. The main ones are summarized in **Table 2**.

### 4.1 Soluble CD163

CD163 is a transmembrane protein expressed on the surface of M2 macrophages and monocytes. In response to inflammatory stimuli, it undergoes proteolytic cleavage and is released as a soluble protein. In the kidney, activated macrophages secrete soluble CD163 (sCD163) into the urine, making it a promising biomarker for active glomerular inflammation.

A recent multicenter study involving 210 AAV patients showed that urinary sCD163 levels were higher in ANCA-positive patients with glomerulonephritis compared to those with extrarenal AAV and patients in remission. This study also demonstrated that urinary sCD163 exhibited high specificity (≥0.96) and reasonable sensitivity (0.69-0.88) for detecting active ANCA-associated glomerulonephritis (154). These findings align with a previous cohort study of patients with small vessel vasculitis (n=177), which established that a urinary sCD163 cut-off of 300 ng/mmol urine creatinine could identify active renal vasculitis with 96% specificity and 83% sensitivity (155). Furthermore, urinary sCD163 concentrations correlated with histological features observed in biopsies from patients with active ANCA glomerulonephritis, reinforcing its potential as a reliable biomarker (154, 156).

Urinary sCD163 levels have also proven effective in detecting disease relapse. In a study of 47 patients with ANCA-associated glomerulonephritis from two independent cohorts, an absolute increase in urinary sCD163 of 20 ng/mmol urine creatinine or a relative increase of 20% from prior measurements identified disease relapse with 100% sensitivity and approximately 90% specificity (157). Additionally, a urinary sCD163 cut-off of 250 ng/mmol urine creatinine successfully differentiated renal vasculitis flares from non-vasculitic acute kidney injury, a common flare mimic (158).

More recently, combining urinary sCD25 (>125 ng/mmol urine creatinine) and serum sCD25 (>1050 pg/ml) with urinary sCD163 (>350 ng/mmol urine creatinine) has shown improved performance in identifying AAV compared to traditional markers and urinary sCD163 alone in detecting active renal vasculitis (sensitivity: 84.7%, specificity: 95.1%) (159).

Additionally, a pilot study (n=45 AAV patients vs 10 healthy subjects) revealed that combining sCD163 with four other urinary proteins (DKK-3, EGF, PRO-C6, C3M) could differentiate AAV patients from controls with 100% accuracy. The biomarker panel also effectively classified biopsy subgroups (focal class vs. other classes: 90% accuracy, AUC 0.964 or 0.925 when serum creatinine was included; focal class vs. crescentic class: 95.2% accuracy; focal class vs. sclerotic class: 95.4% accuracy) (160). Furthermore, biomarker levels at diagnosis predicted final renal function at follow-up with high accuracy (88.9%, AUC: 0.961 or 92.6% when including serum creatinine). However, these findings require validation in a larger cohort, and the lack of longitudinal biomarker assessment highlights the need for further research.

In summary, urinary sCD163 appears to be a promising and reliable biomarker for detecting active disease and predicting relapse in AAV (**Table 2**). However, prospective multicenter studies are required to establish standardized reference values, enabling its use as a diagnostic and prognostic tool in clinical practice.

### 4.2 Monocytes chemoattractant protein-1

Several studies assessing urinary monocyte chemoattractant protein (MCP-1), also known as CCL2, have shown promising results. MCP-1 levels are elevated in patients with active renal ANCA vasculitis and during flares, while they tend to decline in response to treatment.

Urinary MCP-1 concentrations were higher in ANCA-positive patients with active or persistent renal vasculitis compared to healthy controls, as well as in those with extra-renal disease and inactive disease (82, 161–164). Moreover, urinary MCP-1 levels were elevated in patients with severe prognosis compared to those with non-severe prognosis and healthy controls and were associated with worse outcomes (164, 165). These findings highlight the potential of urinary MCP-1 as a biomarker for identifying active renal involvement and poor prognosis in vasculitis. Specifically, a urinary MCP-1 cut-off value > 0.53 ng/ml demonstrated a sensitivity of 100% and a specificity of 75% in distinguishing active from inactive disease (82). Similarly, another study indicated that a 1.3-fold increase in MCP-1 had a sensitivity of 94% and a specificity of 89% in differentiating active renal disease from remission (161).

Furthermore, urinary MCP-1 levels decreased with treatment and improved renal function, correlating with glomerular macrophage infiltration, BVAS, and the renal component of BVAS (162).

Notably, the combination of urinary sCD163 and MCP-1 has proven to be a valuable tool for diagnosing subtle renal vasculitis flares. Both proteins increase during active renal flares, and when integrated into a recursive partitioning tree along with new or worsening proteinuria, they significantly improve the positive likelihood ratio for detecting active renal vasculitis, highlighting their strong clinical potential (166).

### 4.3 Others

**Table 2** summarizes other potential urinary biomarkers. Hematuria and proteinuria are routinely assessed in clinical practice to evaluate renal disease activity in AAV. However, hematuria may have non-glomerular causes, and the lack of standardized methodologies and definitions limits its reliability as a biomarker (167). A recent large retrospective cohort study involving 571 AAV patients demonstrated that proteinuria is an independent predictor of death or kidney failure [uPCR ≥ 0.05 g/mmol, HR: 3.06, 95% CI: 1.09-8.59]. Additionally, both persistent proteinuria [uPCR ≥0.05 g/mmol, HR: 2.2, 95% CI: 1.16-4.24] and hematuria [HR: 2.16, 95% CI: 1.13-4.11] during therapy were identified as independent predictors of kidney relapse (168).

Another retrospective study (n=191) found that MPO-AAV patients with predominant urinary isomorphic red blood cells at diagnosis were more likely to experience severe clinical manifestations and poor renal outcomes (169). However, the retrospective nature of these studies and the inherent fluctuations in proteinuria and hematuria over time were significant limitations.

In addition to CD163 and MCP-1, other molecules have been explored as potential urinary biomarkers in AAV. As mentioned in paragraph 2.3, the activation of the alternative complement pathway plays a crucial role in the pathogenesis of AAV. Urinary levels of Bb, C3a, C5a, and sC5b-9 were found to be higher in patients with active AAV compared to those in remission and healthy controls (90, 170). Furthermore, urinary Bb levels positively correlated with serum creatinine levels and the proportion of total crescents in kidney biopsies while inversely correlating with the proportion of normal glomeruli (90). These associations suggest that urinary Bb levels warrant further investigation as a potential marker of renal injury severity. More recently, urinary Ba levels have been identified as potential predictors of renal flare in AAV. A cut-off of 12.53 ng/mg urine creatinine demonstrated a sensitivity of 76.2% and a specificity of 68.4% (171).

High-mobility group box 1 (HMGB1) is a protein involved in regulating nucleosome stability within the nucleus. Under specific stimuli, it is secreted and acts as a pro-inflammatory mediator (172). Urinary HMGB1 (uHMGB1) levels were found to be elevated in AAV patients with active nephritis compared to healthy controls (173). However, uHMGB1 levels were similar across different AAV subtypes, between patients at disease onset and those experiencing relapse, as well as between MPO- and PR3-ANCA-positive patients (173). Additionally, uHMGB1 concentrations positively correlated with urinary CD+T cells and effector memory T cells, suggesting a possible interplay between HMGB1 and components of adaptive immunity during active nephritis in AAV (163). Overall, HMGB1 may be implicated in the pathogenesis of AAV and associated with disease activity. However, further studies are needed to explore HMGB1 levels in AAV patients without renal involvement.

Another proposed biomarker for disease activity is urinary epidermal growth factor (EGF). Normalized EGF levels were lower in active AAV patients compared to both patients in remission and controls. They were also lower in patients experiencing treatment failure compared to those with a favorable renal response to treatment. Urinary EGF levels exhibited a positive correlation with eGFR and a negative correlation with the degree of interstitial fibrosis and tubular atrophy. Furthermore, urinary EGF/creatinine excretion was associated with a composite outcome of end-stage renal disease or a 30% reduction in eGFR (HR 0.61, 95% CI 0.45 to 0.83, p=0.001) (174).

Endotrophin (ETP), a type VI collagen fragment, plays a role in macrophage chemotaxis, epithelial-to-mesenchymal transition, and tissue fibrosis (175). Urinary ETP correlates with interstitial kidney fibrosis and performs better than urinary DKK-3, a glycoprotein associated with loss of kidney function, in detecting advanced fibrosis (AUC = 0.759, p=0.002) and fibrosis with tubular atrophy [AUC = 0.839 (95% CI 0.701-0.931), p<0.001] (107).

SerpinA3, also known as alpha-1-antichymotrypsin, is a serine protease inhibitor that regulates neutrophil cathepsin G, involved in bacterial killing and tissue remodeling. It is elevated in urine from AAV patients, correlating with renal fibrosis and kidney injury (176). Longitudinal studies are necessary to assess uSerpinA3 trends and its potential induction by other inflammatory kidney processes.

Activin A regulates inflammatory processes, with urinary levels elevated in AAV patients with renal involvement compared to healthy subjects and those without renal involvement. Levels are higher in active disease than in remission and at disease onset compared to relapse (177). Urinary Activin A concentrations decrease with treatment and are elevated in crescentic glomerulonephritis. However, its specificity remains unclear, and the potential influence of urinary tract infections on its levels requires further investigation.

Gremlin, a TGFβ-induced protein involved in epithelial-to-mesenchymal transition, is an emerging biomarker for crescentic glomerulonephritis in AAV patients (178). A urinary gremlin cut-off of 241 µg/g creatinine [AUC: 0.81, CI 95% 0.68-0.94, p< 0.001] shows promise for detecting renal vasculitis, with 55% sensitivity and 100% specificity. It is highly expressed in glomerular crescents, tubular cells, and interstitial inflammatory cells (179).

Urinary T cells have been explored as biomarkers for identifying active renal AAV and predicting disease outcomes (180). A threshold of 3,149 CD3+ T cells per 100 ml (sensitivity: 90%, specificity: 92%) and 60 Treg cells per 100 ml (sensitivity: 74%, specificity: 96%) identified active renal AAV. Additionally, 200 Treg cells and 250 Th17 cells per 100 ml predicted an early clinical response. For relapse prediction, a combination of > 10 Th17 cells and 0 Treg cells per 100 ml predicted renal flares at 6 months (positive predictive value: 50%; negative predictive value: 100%). However, the small sample size (n=16) limits conclusions, requiring validation in larger cohorts. A recent prospective multicenter study (PRE-FLARED) reported that CD4+ T cells >490 per 100 ml predicted renal relapse within 6 months (sensitivity: 60%, specificity: 97.8%, PPV: 75.2%, NPV: 95.7%) (181).

Other urinary biomarkers under investigation include urinary IgM (uIgM) and urinary mitochondrial DNA (umtDNA). High uIgM concentrations have been associated with poor prognosis in ANCA-associated renal vasculitis, with an odds ratio of 19.8 for end-stage renal disease (182).

UmtDNA levels are elevated in AAV patients with kidney injury and correlate with severe kidney dysfunction. Higher urinary mtDNA levels are observed in the crescentic pathological class and are associated with neutrophil infiltration in kidney biopsies (183). These findings suggest potential roles for uIgM and umtDNA in disease prognosis and severity assessment.

Urinary matrix metalloproteinases (MMPs) and metabolites have been explored as biomarkers of renal damage in AAV. MMP-2, MMP-9, and TIMP-1 levels were elevated in AAV patients compared to controls. MMP-2 activity and TIMP-1 levels correlated with tubulointerstitial fibrosis and atrophy and were negatively associated with creatinine clearance (184).

Regarding urinary metabolites, the myo-inositol:citric acid ratio was significantly higher in patients with active renal vasculitis compared to controls and those in remission, effectively detecting active renal vasculitis [AUC:0.922, 95% CI 0.849-0.970, p=0.001)] (185). A recent small study (n=10) also found that citrate and iso-citrate levels were lower in urine samples from active AAV patients compared to those in remission (186). These findings highlight the potential of urinary MMPs and metabolites as indicators of disease activity and kidney damage in AAV.

Considering epigenetics, microRNAs (miRNAs) regulate gene expression post-transcriptionally and have been studied for their role in AAV pathogenesis and as potential biomarkers. Urinary extracellular vesicle (EV) miRNA profiling identified miR-30a-5p, miR-31-3p, miR-99a-5p, miR-106b-5p, and miR-182-5p as significantly elevated in AVV patients compared to controls (187). Integrated miRNA and proteomic analysis in urinary EVs suggested that proteins involved in actin cytoskeleton organization may contribute to AAV pathogenesis (187, 188). Despite these promising findings, the study was conducted on a small cohort, and larger prospective studies are needed to validate the diagnostic potential of these miRNAs in detecting active AAV.

## 5 Future directions and challenges in biomarker validation

**Figure 1** depicts all the available biomarkers and their current or potential role for assessing disease activity, relapse, prognosis or specific organ involvement. However, most of them do not yet provide a comprehensive assessment of disease activity, damage, and prognosis, either individually or within a biomarker panel. While they show promise, validation in independent cohorts and prospective studies are necessary before they can be implemented in daily clinical practice.

Overall, the most promising novel biomarkers for clinical implementation are urinary sCD163 and MCP-1. Urinary sCD163 has been found to be associated with active ANCA-associated glomerulonephritis and specific histological features in kidney biopsies. Therefore, measuring its levels could be useful in clinical practice for diagnosis and monitoring disease activity during patient follow-up, potentially reducing the need for kidney biopsies. Biomarker cut-off values depend on the assay kits used for measurement; thus, this should be considered when comparing different studies. Concerning sCD163, an accredited, validated *in vitro* diagnostic assay has been developed by Euroimmun GMBH. A cut-off of 250 ng/mmol urine creatinine may be used to distinguish active renal AAV from remission and to identify patients with or without flares (158). Regarding MCP-1, based on the Quantikine ELISA from R&D, a cutoff of 0.53 ng/ml can differentiate between active and remission AAV patients with kidney involvement (82).

In clinical practice, we already utilize levels of ANCA, including MPO and PR-3, CRP and ESR, CD19^+^ B cells, and complement levels to assess disease activity. In the future, with the addition of more biomarkers, biomarker panels to evaluate different aspects of the disease would be beneficial. In the era of precision medicine, this would allow assessing the patient’s risk of experiencing a more relapsing disease or developing specific organ involvement.

Ongoing research is using advanced analytical techniques to simultaneously investigate multiple biomarkers, improving the understanding of disease activity, progression, and prognosis in AAV. Approaches such as proteomics, transcriptomics, and metabolomics allow for a comprehensive assessment of potential biomarkers and support their validation for clinical use. Proteomics, in particular, offers a multiplex approach for profiling proteins at a specific time point, helping to differentiate active AAV from remission (189).

Biospectroscopy is an emerging method that utilizes spectroscopic techniques to analyze macromolecular classes (proteins, lipids, carbohydrates, and nucleic acids) within individual cells through a single global measurement (190). This approach enables the simultaneous identification of biochemical changes in tissue associated with disease states, aiding in biomarker discovery and automated lesion detection. Spectral data can be obtained from various biological samples, including urine or blood, offering a potential non-invasive alternative to renal biopsy. Comparing urine samples with renal biopsy findings could help explore the potential of biospectroscopy as a liquid biopsy for assessing histological activity in renal vasculitis. This approach would offer a non-invasive method for evaluating disease progression and activity. While a recent study has begun to investigate this potential, larger validation studies are needed to confirm its clinical utility and establish its reliability as a diagnostic tool (191).

Machine learning models are already providing valuable insights into the diagnosis and classification of AAV. Specifically, subclassifying AAV based on prominent organ involvement has shown a higher predictive value for key outcomes (192, 193). This approach allows for more tailored and accurate assessments of disease severity and progression. Looking ahead, deep learning models could play a critical role in screening and comparing current methods for assessing disease activity and damage in AAV. Additionally, these models could help identify potential prognostic indicators, offering more precise tools for clinical decision-making and personalized treatment strategies.
